# Age-dependent plasticity in the superior temporal sulcus in deaf humans: a functional MRI study

**DOI:** 10.1186/1471-2202-5-56

**Published:** 2004-12-08

**Authors:** Norihiro Sadato, Hiroki Yamada, Tomohisa Okada, Masaki Yoshida, Takehiro Hasegawa, Ken-Ichi Matsuki, Yoshiharu Yonekura, Harumi Itoh

**Affiliations:** 1 National Institute for Physiological Sciences, 38 Nishigonaka, Myodaiji, Okazaki, 444-8585, Japan; 2 JST/RISTEX, 2-5-1, Atago, Minato-ku, Tokyo, 105-6218, Japan; 3 Fukui University School of Medicine, Fukui, 910-1193, Japan; 4 Fukui University School of Education, Fukui, 910-1193, Japan; 5 Biomedical Imaging Research Center, Fukui University School of Medicine, Fukui, 910-1193, Japan

## Abstract

**Background:**

Sign-language comprehension activates the auditory cortex in deaf subjects. It is not known whether this functional plasticity in the temporal cortex is age dependent. We conducted functional magnetic-resonance imaging in six deaf signers who lost their hearing before the age of 2 years, five deaf signers who were >5 years of age at the time of hearing loss and six signers with normal hearing. The task was sentence comprehension in Japanese sign language.

**Results:**

The sign-comprehension tasks activated the planum temporale of both early- and late-deaf subjects, but not that of hearing signers. In early-deaf subjects, the middle superior temporal sulcus was more prominently activated than in late-deaf subjects.

**Conclusions:**

As the middle superior temporal sulcus is known to respond selectively to human voices, our findings suggest that this subregion of the auditory-association cortex, when deprived of its proper input, might make a functional shift from human voice processing to visual processing in an age-dependent manner.

## Background

There is evidence that cross-modal plasticity induced by auditory deprivation is apparent during sign-language perception. Sign languages involve the use of the hands and face, and are perceived visually [1-3]. Using functional MRI (fMRI), Neville *et al. *[1] observed increased activity in the superior temporal sulcus (STS) during the comprehension of American Sign Language (ASL) in both congenital deaf subjects and hearing native signers. The authors therefore suggested that the STS is related to the linguistic analysis of sign language. Nishimura *et al. *[2] found that activity was increased in the auditory-association cortex but not the primary auditory cortex of a prelingual-deaf individual during the comprehension of Japanese sign language (JSL). After this patient received a cochlear implant, the primary auditory cortex was activated by the sound of spoken words, but the auditory association cortex was not. The authors suggested that audio-visual cross-modal plasticity is confined to the auditory-association cortex and that cognitive functions (such as sign language) might trigger functional plasticity in the under-utilized auditory-association cortex. In addition, Pettito *et al. *[3] observed increased activity in the superior temporal gyrus (STG) in native deaf signers compared with hearing non-signers. These findings suggest that the changes associated with audio-visual cross-modal plasticity occur in the auditory-association cortex. However, the age dependency of this plasticity is not known. To depict the age dependency of the cross-modal plasticity, we conducted a functional MRI study of deaf signers with both early and late deafness, as well as hearing signers, performing a sign-comprehension task. 'Early deaf' subjects were defined as those who lost their ability to hear before the age of 2 years, whereas 'late deaf' subjects lost their hearing after the age of 5 years.

## Results

**Figure 1:**
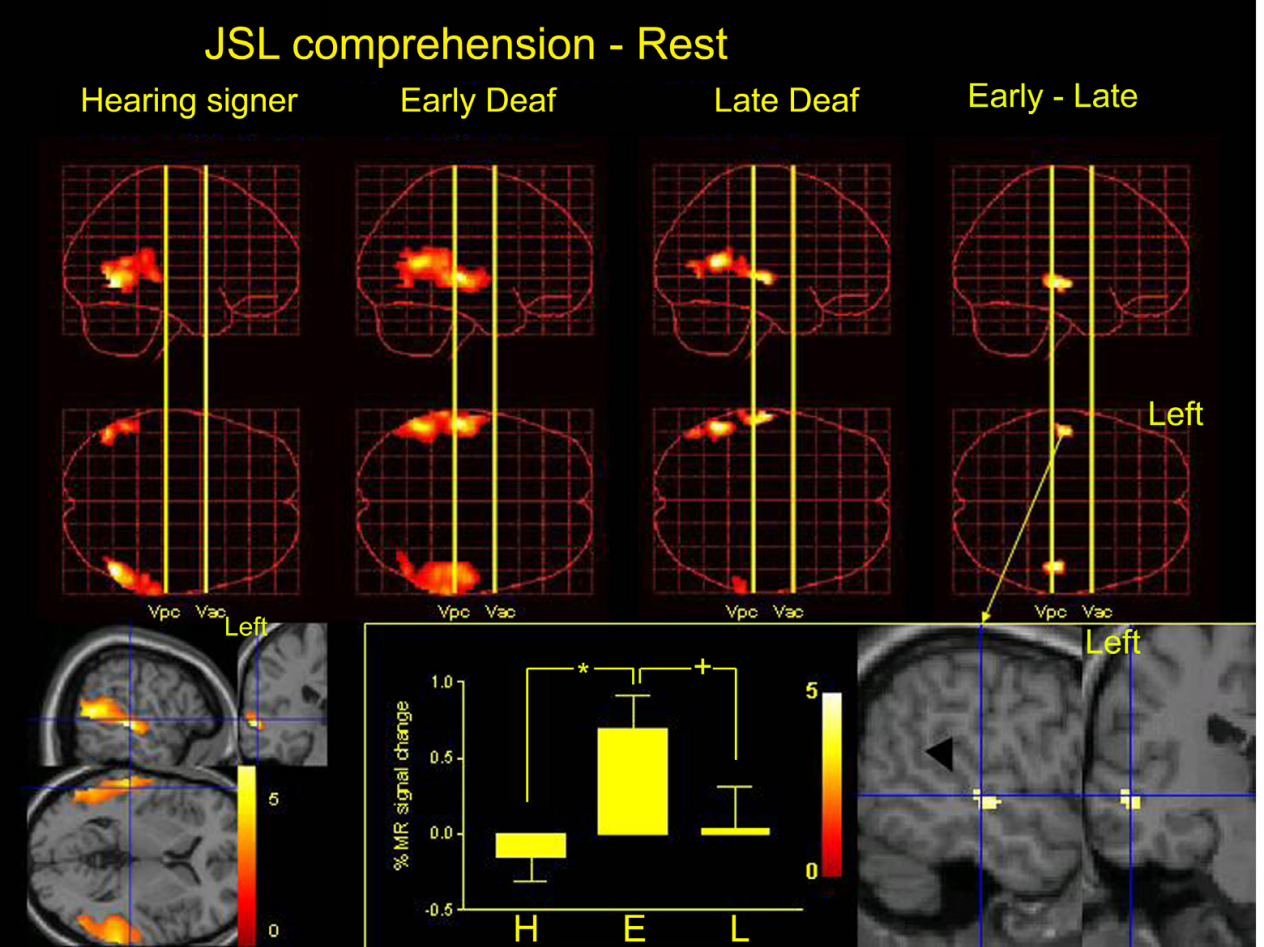
The results of group analysis. Statistical parametric maps of the average neural activity during JSL comprehension compared with rest are shown in standard anatomical space, combining hearing signers (left column), early-deaf signers (Early Deaf; second column) and late-deaf signers (Late Deaf; third column). The region of interest was confined to the temporal cortex bilaterally. The three-dimensional information was collapsed into two-dimensional sagittal and transverse images (that is, maximum-intensity projections viewed from the right and top of the brain). A direct comparison between the early- and late-deaf groups is also shown (E – L, right column). The statistical threshold is P < 0.001 (uncorrected). Right bottom, the group difference of the task-related activation (E – L) was superimposed on sagittal and coronal sections of T1-weighted high-resolution MRIs unrelated to the subjects of the present study. fMRI data were normalized in stereotaxic space. The blue lines indicate the projections of each section that cross at (-52, -22, -2). The black arrowhead indicates the STS. Bottom middle, the percent MR signal increase during JSL comprehension compared with the rest condition in the STS (-52, -22, -2) in hearing signers (H), early-deaf (E) and late-deaf signers (L). There was a significant group effect (F(2, 14) = 23.5, P < 0.001). * indicates P < 0.001, + indicates P = 0.001 (Scheffe's post hoc test). Bottom left, task-related activation in the deaf (early + late) groups. The blue lines indicate the projections of each section that cross at (-56, -26, 4). In the deaf subjects, the superior temporal cortices are extensively activated bilaterally.

Performance on the JSL comprehension task was similar across the groups (F(2, 14) = 1.279, P = 0.309, one-way ANOVA). The patterns of activity evoked during the sign-comprehension task in the hearing signers and the deaf groups are shown in Figure 1. Within the temporal cortex, all groups showed activation in the occipito-temporal junction extending to the portion of the STG posterior to the Vpc line (an imaginary vertical line in the mid-sagittal plane passing through the anterior margin of the posterior commissure). In the early- and late-deaf subjects, the activation of the posterior STG extended anteriorly to the Vpc line to reach the Vac line (an imaginary vertical line in the mid-sagittal plane passing through the posterior margin of the anterior commissure). The activation was confined to the STG, extending into the superior temporal sulcus, and was more prominent on the left side. A direct comparison between early- and late-deaf subjects revealed significantly more prominent activation of the bilateral middle STS in the early-deaf subjects (Figure 1).

## Discussion

The onset of deafness is related to language acquisition. Prelingual deafness occurs before spoken language is learned. Hearing people generally learn their first language before 5 years of age; hence, prelingual deaf individuals are either deaf at birth or became deaf prior to developing the grammatical basis of their native language, which is usually before the age of 5 years. Postlingual deafness is the loss of acoustic senses, either suddenly due to an accident or as a gradual progression after native-language acquisition [4]. Hence, the early-deaf subjects in the present study are categorized as 'prelingual deaf' and the late-deaf subjects are categorized as 'postlingual deaf'. More than 90% of children with prelingual hearing loss have parents with normal hearing [5]. Furthermore, in Japan, the traditional teaching method for deaf children includes aural/oral methods, such as lipreading. Native signers are usually limited to those who were brought up by deaf parents. Because of this, the majority of prelingual deaf subjects learn spoken language (Japanese) in artificial ways, such as aural/oral methods. In the present study, the parents of the deaf subjects all had normal hearing. Five out of six of the early-deaf subjects started JSL training after the age of 6 years. Thus, JSL is not the first language for any of the groups in the present study.

The posterior STS was activated in all groups during sign comprehension, which is consistent with the proposed neural substrates that subserve human movement perception [6]. The posterior STS region is adjacent to MT/V5, which is consistently activated during the perception of human body movement [7-9]. Hence, the activation of the posterior STS in both hearing and deaf subjects is related to the perception of the movement of the hands and mouth.

Both the early- and late-deaf groups showed activation in the planum temporale, whereas hearing signers did not. Anatomically, the anterior border of the PT is the sulcus behind Heschl's gyrus and the medial border is the point where the PT fades into the insula. The posterior border of the PT involves the ascending and descending rami of the Sylvian fissure [10]. Functionally, the left PT is involved in word detection and generation, due to its ability to process rapid frequency changes [11,12]. The right homologue is specialized for the discrimination of melody, pitch and sound intensity [13,14].

It has been shown that non-linguistic visual stimuli (moving stimuli) activate the auditory cortex in deaf individuals, but not in hearing subjects [15,16]. McSweeney *et al. *[17] showed that the planum temporale is activated in deaf native signers in response to visual sign-language images and this activation is larger for native deaf signers compared to hearing signers. Our previous study [18] revealed that cross-modal activation in the temporal cortex of the deaf subjects was triggered not only by signs but also by non-linguistic biological motion (lip movement) and non-biological motion (moving dots). Signs did not activate the temporal cortex of either the hearing signers or the hearing non-signers. Thus, in the present study, the activation of the planum temporale in the early- and late-deaf subjects is probably due to the effects of auditory deprivation, rather than linguistic processes. This theory is also supported by the fact that the hearing signers in the present study did not show temporal-lobe activity during JSL comprehension, whereas the PT was more prominently activated in the deaf subjects irrespective of the timing of the onset of deafness. These findings indicate that auditory deprivation plays a significant role in mediating visual responses in the auditory cortex of deaf subjects. This is analogous with findings related to visual deprivation: irrespective of the onset of blindness, the visual-association cortex of blind subjects was activated by tactile-discrimination tasks [19,20] that were unrelated to learning Braille [20]. These results suggest that the processing of visual and tactile stimuli is competitively balanced in the occipital cortex. A similar competitive mechanism might occur in the PT following auditory deprivation. Activation of the STG in hearing subjects during lipreading [21] indicates which cortico-cortical circuits might be involved in the competitive balance between the modalities. In fact, we found that the cross-modal plasticity in the deaf subjects occurred within the neural substrates that are involved in lipreading in hearing subjects [18].

The middle STS, anterior to the Vpc line, was activated more prominently in the early- than the late-deaf subjects. This difference is probably not related to linguistic processes, as both early- and late-deaf subjects are equally capable of learning JSL with the same amount of training. The middle STS region is presumably the area that is selective to human voice processing [22]. This area is known to receive predominantly auditory input, being involved in the high-level analysis of complex acoustic information, such as the extraction of speaker-related cues, as well as the transmission of this information to other areas for multimodal integration and long-term memory storage [22]. This implies that early auditory deprivation (at <2 years of age) might shift the role of the middle STS from human voice processing to the processing of biological motion, such as hand and face movements (cross-modal plasticity). It has been suggested that once cross-modal plasticity occurs in the auditory cortex, the restoration of auditory function by means of cochlear implants is ineffective [23]. Hence, the first 2 years of life might be the sensitive period for the processing of human voices.

Considering that the STS voice-selective area is not sensitive to speech *per se *but rather to vocal features that carry nonlinguistic information [22], the functional role of this region in early-deaf subjects with regard to the paralinguistic aspects of sign language is of particular interest and further investigation will be necessary.

## Conclusions

The results of the present study suggest that in early-deaf subjects, non-auditory processing, such as that involved in the perception and comprehension of sign language, involves the under-utilized area of the cortex that is thought to be selective to the human voice (middle STS). This indicates that the sensitive period for the establishment of human voice processing in the STS might be during the first 2 years of life.

## Methods

The subjects comprised six early-deaf signers (mean age: 22.8 ± 3.1 years), five late-deaf signers (mean age: 34.4 ± 16.2 years) and six hearing signers (mean age: 33.7 ± 12.1 years; Table 1). The early-deaf subjects lost their hearing before 2 years of age, whereas the late-deaf subjects became deaf after the age of 5 years. The parents of all subjects had normal hearing. None of the subjects exhibited any neurological abnormalities and all had normal MRI scans. None of the cases of deafness were due to a progressive neurological disorder. All deaf and hearing subjects were strongly right handed, except for one late-deaf subject who was ambidextrous, according to the Edinburgh handedness inventory [24]. The study protocol was approved by the Ethical Committee of Fukui University School of Medicine, Japan, and all subjects gave their written informed consent.

The tasks involved the passive perception of JSL sentences that are frequently used in the deaf community. JSL, which has its own grammar, morphemes and phonemes, is different from spoken Japanese at all levels. JSL utilizes facial expressions as obligatory grammatical markers, as does ASL [25]. The fMRI session with JSL consisted of two rest and two task periods, each of 30 seconds duration, with alternating rest and task periods. During the 30-second task period, the subjects were instructed to observe a JSL sentence presented every 5 seconds by a male deaf signer in a video, which was projected onto a screen at the foot of the scanner bed and viewed through a mirror. The sentences were relatively short and straightforward; for example, "I cut a piece of paper with scissors". During the 30-second rest period, the subjects fixed their eyes on the face of a still image of the same person. Each session started with a rest period and two fMRI sessions were conducted. The procedure was identical for all hearing and deaf subjects. After the fMRI session, outside of the scanner, the subjects were presented the JSL sentences used during the session. These were shown one by one on the video screen and the subjects were required to write down the presented sentences in Japanese. On each presentation, the subjects were asked if they had seen the JSL sentence in the scanner, in order to confirm that they had been engaged in the task during the session. The percentage of correct responses was calculated as the number of correctly written sentences divided by the number of presented sentences.

A time-course series of 43 volumes was produced using T2*-weighted gradient-echo EPI sequences with a 1.5 Tesla MR imager (Signa Horizon, General Electric, Milwaukee, Wisc., USA) and a standard birdcage head coil. Each volume consisted of 11 slices, with a slice thickness of 8 mm and a 1-mm gap, which covered the entire cerebral cortex. The time interval between two successive acquisitions of the same image was 3,000 ms, the echo time was 50 ms and the flip angle was 90 degrees. The field of view was 22 cm. The digital in-plane resolution was 64 × 64 pixels. For anatomical reference, T1-weighted images were also obtained for each subject.

The first three volumes of each fMRI session were discarded because of unstable magnetization. The remaining 40 volumes per session were used for statistical parametric mapping (SPM99, Wellcome Department of Cognitive Neurology, London, UK) implemented in Matlab (Mathworks, Sherborn, Mass., USA) [26,27]. Following realignment and anatomical normalization, all images were filtered with a Gaussian kernel of 10 mm (full width at half maximum) in the *x*, *y *and *z *axes.

Statistical analysis was conducted at two levels. First, the individual task-related activation was evaluated. Second, the summary data for each individual were incorporated into the second-level analysis using a random-effects model to make inferences at a population level. The signal was proportionally scaled by setting the whole-brain mean value to 100 arbitrary units. The signal time course for each subject was modeled using a box-car function convolved with a hemodynamic-response function and temporally high-pass filtered. Session effects were also included in the model. The explanatory variables were centered at zero. To test hypotheses about regionally-specific condition effects (that is, sentence comprehension compared with rest), estimates for each model parameter were compared using the linear contrasts. The resulting set of voxel values for each contrast constituted a statistical parametric map (SPM) of the *t *statistic (SPM{*t*}).

The weighted sum of the parameter estimates in the individual analyses constituted 'contrast' images that were used for the group analysis. Contrast images obtained via individual analyses represent the normalized task-related increment of the MR signal of each subject. To examine group differences (prelingual deaf, postlingual deaf and hearing signers) in activation due to the sign-comprehension task, a random-effect model was performed with the contrast images (1 per subject) for every voxel. Using the *a priori *hypothesis that there would be more prominent activation in the early- than late-deaf subjects, we focused on the temporal cortex, which was anatomically defined in standard stereotaxic space [28]. The threshold for SPM{*t*} was set at *P *< .001 without a correction for multiple comparisons.

## Authors' contributions

NS carried out the fMRI studies, data analysis and drafted the manuscript. HY and TO conducted the MR imaging. MY, TH and KM prepared the task materials. YY and HI participated in the task design and coordination. All authors read and approved the final manuscript.

## Figures and Tables

**Table 1:** Subject profiles

						Lost audition (dB)		
	Age (years)	Sex	Age of deafness onset (years)	Age of beginning JSL training (years)	Duration of JSL training	Right ear	Left ear	Performance (% correct)
Early-deaf signers								
1	27	M	0	20	7	95	95	83.3
2	20	M	0	3.5	16.5	90	90	66.7
3	24	M	0	14	10	71	86	75.0
4	19	M	0	6	13	100	100	58.3
5	22	M	0	7	15	95	95	66.7
6	25	M	2	19	6	90	90	66.7
Average	22.8		0.3	11.58	11.3	90.2	92.7	69.4
Late-deaf signers								
7	21	F	9	11	10	100	100	83.3
8	22	M	5	6	16	120	120	66.7
9	35	F	10	20	15	120	120	100.0
10	61	F	11	55	6	90	120	58.3
11	33	F	6	27	6	90	90	91.7
Average	34.4		8.2	23.8	10.6	104	110	80.0
Hearing signers								
12	46	M		25	21			75.0
13	40	F		32	8			66.7
14	26	F		24	2			83.3
15	20	F		16	4			75.0
16	23	F		21	2			83.3
17	47	F		27	20			75.0
Average	33.7			24.2	9.5			76.4
